# Genetic Engineering of Microalgae for Secondary Metabolite Production: Recent Developments, Challenges, and Future Prospects

**DOI:** 10.3389/fbioe.2022.836056

**Published:** 2022-03-23

**Authors:** Arathi Sreenikethanam, Subhisha Raj, Rajesh Banu J, Poornachandar Gugulothu, Amit K. Bajhaiya

**Affiliations:** ^1^ Algal Biotechnology Lab, Department of Microbiology, School of Life Sciences, Central University of Tamil Nadu, Thirvarur, India; ^2^ Department of Biotechnology, Central University of Tamil Nadu, Thirvarur, India

**Keywords:** microalgae, high value products, secondary metabolites, genetic engineering, transformation, synthetic biology

## Abstract

Microalgae are highly diverse photosynthetic organisms with higher growth rate and simple nutritional requirements. They are evolved with an efficiency to adapt to a wide range of environmental conditions, resulting in a variety of genetic diversity. Algae accounts for nearly half of global photosynthesis, which makes them a crucial player for CO_2_ sequestration. In addition, they have metabolic capacities to produce novel secondary metabolites of pharmaceutical, nutraceutical and industrial applications. Studies have explored the inherent metabolic capacities of microalgae with altered growth conditions for the production of primary and secondary metabolites. However, the production of the targeted metabolites at higher rates is not guaranteed just with the inherent genetic potentials. The strain improvement using genetic engineering is possible hope to overcome the conventional methods of culture condition improvements for metabolite synthesis. Although the advanced gene editing tools are available, the gene manipulation of microalgae remains relatively unexplored. Among the performed gene manipulations studies, most of them focus on primary metabolites with limited focus on secondary metabolite production. The targeted genes can be overexpressed to enhance the production of the desired metabolite or redesigning them using the synthetic biology. A mutant (KOR1) rich in carotenoid and lipid content was developed in a recent study employing mutational breeding in microalgae (Kato, Commun. Biol, 2021, 4, 450). There are lot of challenges in genetic engineering associated with large algal diversity but the numerous applications of secondary metabolites make this field of research very vital for the biotech industries. This review, summarise all the genetic engineering studies and their significance with respect to secondary metabolite production from microalgae. Further, current genetic engineering strategies, their limitations and future strategies are also discussed.

## Introduction

Microalgae are eukaryotic organisms that have evolved through a series of primary and secondary endosymbiosis (Fu et al., 2019). They are ubiquitous in nature and are adapted to cope with a wide range of environmental conditions. Microalgae are regarded as most promising feed-stocks for metabolite production due to its higher growth rate, biomass production and ease of cultivation. However, improved productivity is dependent on a number of parameters including light intensity and nutrient requirements, which varies with species and the target metabolite (Gimpel et al., 2015). Microalgae are taxonomically diverse and have complex metabolic pathways that provide a source for a variety of value-added molecules which can be used in commercial production of antimicrobials, photo protectants, bioplastics (Bajhaiya, 2021), antivirals etc. (Fu et al., 2019). These molecules are lipids, carbohydrates, or secondary metabolites that can be applied in a bio-refinery approach to produce commercially viable pharmaceutical, nutraceutical, or cosmeceutical products. However, under natural conditions, the rate of metabolite production inside the cell is insufficient for large-scale production. Moreover, metabolite composition changes with the growth stages to meet the needs of the cell. Although, microalgae have a simple organisation and genetic makeup compared to plants, but their enormous diversity and genetic stability make it difficult to develop advanced molecular tools for biotechnological applications. Once advanced techniques have been developed, the next challenge is the delivery of foreign genetic material into algal cells. Various gene delivery systems are used to accomplish this. However, because most genome editing research is done in simple species like bacteria and yeasts, microalgae do not have exposure to all of the existing gene delivery mechanisms (Gutiérrez and Lauersen, 2021).

Just like plants, carbohydrates and other primary metabolites are required for cell development and division in microalgae. This is especially more important in the early phases of the growth, when the algal cells are actively growing. Secondary metabolites are usually produced near the end of the growth cycle, when the organism is approaching the end of life cycle. This is because cells will be encountered with stress due to environmental factors such as nutrient availability and toxin exposure at this stage. These secondary metabolites have features that assist cells in coping with stress and initiate certain signalling pathways involved in the defence responses (Jeong et al., 2020). Up till now the intrinsic ability of microalgae to produce and accumulate significant amounts of secondary metabolites under stress conditions is used. Therefore, as of now there are not many secondary metabolites of algal origin, which are commercialized. Due to various benefits of algal metabolites, the focus has recently shifted to algal genome editing, and several algal strains are thoroughly investigated for their possible biotechnological applications (Gutiérrez and Lauersen, 2021). Currently, stress responses are mostly studied through “omic” analysis to identify the major regulators of metabolite production pathways, and the genetic manipulations are carried out to increase the yields (Guihéneuf et al., 2016). Usually the genetic alterations are performed with an objective of increasing target metabolite production. But, sometime foreign genes, either nuclear or plastid genomes, can also be acquired naturally from other species via horizontal gene transfer (Gutiérrez and Lauersen, 2021). Similarly the metabolic drifts are also reported in evolution studies (Koch et al., 2017; Belcour et al., 2020). Bioinformatic tools are efficiently used to identify these types of transfers by comparing homologues in the relevant genome sequences. Further, combining algal engineering with advanced technologies like Artificial Intelligence (AI) will help in prediction of molecular interactions and their products (Yong et al., 2020). In addition, active metabolic profiling and comparison can also help in discovering the novel secondary metabolites of commercial importance.

Microalgae can serve as a sustainable feedstock for production of several secondary metabolites to meet the requirements of growing population. These metabolites can be of pharmaceuticals, cosmeceuticals or nutraceuticals applications. In particular, the wide spectrum of applications are due to their key features such as antioxidant, anti-inflammatory, antimicrobial, hepatoprotective, neuroprotective and anticancer properties (Jeong et al., 2020). Here we have discussed in detail the microalgal secondary metabolites and their industrial applications. Furthermore, the significance of algal genetic engineering, various gene delivery systems, current approaches, challenges, possible future strategies, along withkey findings is critically analysed and discussed.

## Microalgal Secondary Metabolites

Microalgae are considered as unique phototrophic cell factories, which can convert CO_2_ and carbon containing organic substance into primary and secondary metabolites. The primary metabolites (Carbohydrates, proteins and, lipids) are key compounds, which are essential for the survival of the organism (Vuppaladadiyam et al., 2018). The secondary metabolites are compounds (pigments, alkaloids, isoprenoids, mycosporine-like amino acids (MAAs), sterols), which are required for the auxiliary purpose, such as defence mechanism, stress responses, signalling, etc. and are produced in the idiophase of the growth cycle (Jeong et al., 2020). It is important to understand the structure and properties of these secondary metabolites to utilize them at industrial level. Below different classes of secondary metabolites produced by microalgae are discussed with their known applications and further summarized in Table 1.

**TABLE 1:** Microalgal secondary metabolites and its applications.

Class	Secondary metabolite	Organism	Percentage (dry weight)	Applications	References
Carotenoids	β-carotene	*Dunaliella salina, Spirulina maxima,* Dunaliella bardawil	0.2–1%	Anti-inflammatory, Anti-oxidant, food colorant, Vitamin A precursor, Reduces macular degeneration and colorectal cancer risk	(Bule et al., 2018)
	—	—	—	—	
	Lutein	*Dunaliella salina, Chlorella pyrenoidosa, Chlorella protothecoids,* Chlorella sorokiniana, Chlorella vulgaris	0.5–1.2%	Prevents age-related cataract and maculopathy, anti-tumor, food coloring agent (egg-yolk coloring agent), feed additive, anti-oxidant	(Bule et al., 2018)
	—	—	—	—	
	Astaxanthin	*Haematococcus pluvialis, Chlorella zofigiensis, Chlorella vulgaris, Chlorococcum* sp	1–8%	Anti-inflammatory, Anti-oxidant, anti-tumour, food coloring agent, poultry and aquaculture feed additive, anti-ageing, sun protection, anti-diabetic	
	—	—	—	—	
	—	—	—	—	Bule et al. (2018)
	Violaxanthin	*Dunaliella tertiolecta, Chlorella ellipsoidea*	—	Anti-inflammatory, Anti-tumor	(Bule et al., 2018)
	—	—	—	—	
	Zeaxanthin	*Dunalilella salina, P. cruentum*, Chlorella saccharophila Synechocystis sp., Microcystis *aeruginosa*, Nannochloropsis oculate	1–2%	Anti-inflammatory, Anti-oxidant, prevents age related cataract, reduces macular degeneration	
	—	—	—	—	
	—	—	—	—	Bule et al. (2018)
	Fucoxanthin	Phaeodactylum tricornutum, Isochrysis sp	>1.5%	Anti-inflammatory, Anti-oxidant, anti-cancer, anti-obesity, prevents cerebrovascular diseases	(Bule et al., 2018)
Polyunsaturated Fatty Acids (PUFAs)	—	*Chlorella vulgaris, Tetraselmis* sp.*,* Nannochloropsis oculate, *Crypthecodinium conhi, Nannochloropsis gaditana, Schizochytrium* sp.*, Ulkenia* sp	—	Role in neurogenesis and neurotransmission, treatment of various diseases (cancer, atherosclerosis, rheumatoid arthritis, Alzheimer’s, and psoriasis), anti-inflammatory, nutrition supplements	(Bule et al., 2018)
	—	—	—	—	
	—	—	—	—	
Tocopherols	—	Porphydium sp., Spirulina platensis, Desmodesmus sp.	—	Vitamin-E antioxidant activity, anti-inflammatory, anti-tumoural	
	—		—		
phenolic compounds	hydroxycinnamic acids	*Chlorella vulgaris, Haematococcus pluvialis, Diacronema lutheri, Phaeodactylum* sp*., Tetraselmis suecica, Porphyridium purpureum*	—	Anti-oxidant, anti-inflammatory	
	4-hydroxybenzaldehyde	Spongiochloris spongiosa, Spirulina platensis, Anabaena doliolum, Nostoc *sp*., Cylindrospermum sp.	—	—	
	3,4-dihydroxybenzaldehyde	Spongiochloris spongiosa, Spirulina platensis, Anabaena doliolum, Nostoc sp., Cylindrospermum sp.	—	—	—
	Vanillic/syringic/caffeic/chlorogenic acid	Spirulina sp.	—	—	—
Phycobiliproteins	phycocyanin	*Oscillatoria agardhii, Nostoc UAM 206, Spirulina platensis, Galdiera suphuraria*	About 20%	food and cosmetics coloring agent, fluorescence immunoassays reagent, anti-oxidant	
	phycoerythrin	*Nostoc UAM 206, Spirulina platensis, Phorphyridium aerugineum, Porphyridium cruentum*	—	fluorescence immunoassays reagent, label for biological molecules	—
Mycosporine like Amino acids		*Nostoc commune, Alexandrium* sp. (*Alexandrium tamarense, A. catenella and A. minutum*)*, Lyngbya* sp.*, Synechocystis* sp., *Chlorella sorokiniana, Calothrix* sp	—	Sunscreen, anti-inflammatory, anti-tumour, anti-oxidative, wound healing	(Llewellyn, n.d
			—		Raj et al., 2021)
			—		
scytonemin	—	*Fischerella muscicola, Nostoc commune, Chlorogloeopsis* sp., *Scytonema* sp.*, Nostoc punctiforme*	—	Anti-inflammatory, Anti-proliferation, sun protection	(Llewellyn, n.d.)
	—	—	—	—	

### Bio-Pigments

Microalgae consist of three major classes of bio-pigments *viz.,* chlorophylls, carotenoids and accessory pigments like phycobilins. These bio-pigments are light-absorbing substances that absorb light in a variety of visible spectrum wavelengths (Chakdar and Pabbi, 2017). The backbone of these bio-pigments consists of tetrapyrrole rings primarily in chlorophyll and phycobilins and isoprene units in carotenoids (Saini et al., 2020). Chlorophyll is the major light-harvesting pigment in microalgae (Gan and Bryant, 2015). The chlorophyll structure has four pyrrole rings arranged in a tetrapyrrole ring, which referred as porphyrin, a tetrapyrrole ring structure (Saini et al., 2020).

Carotenoids are light harvesting pigments predominately found with chlorophylls. They primarily absorb electromagnetic radiation in the region of the visible spectrum, which is not absorbed by the chlorophyll (Cerezo et al., 2012). The structure of carotenoids is formed by 40 carbon isoprene unit and are divided into different groups by the presence or absence of oxygen at the terminal end (Vuppaladadiyam et al., 2018). The derivatives, which is non-oxygenated are called carotene, and their oxygenated derivatives are xanthophylls (Sabarinathan and Gomathy, 2011). Carotenoids are well-known for its antioxidant and anticancer properties. The different types of carotenoids produced by microalgae contains β-carotene, astaxanthin, lutein, lycopene, canthaxanthin and Fucoxanthin, etc (Chakdar and Pabbi, 2017). *Chlorella zofingiensis, Chlorella vulgaris, Dunaliella salina* and *Haematococcus pluvialis* are some of the common species employed in commercial production of carotenoids (Vuppaladadiyam et al., 2018). Although, secondary carotenoids like β-carotene are produced at high levels at stress conditions, primary carotenoids like lutein, when exposed to high stress conditions can undergo degradation. Astaxanthin synthesis is dependent on several factors such as nutrient availability, salt concentration, heavy metal content, nitrogen source, light quality and intensity of light. Factors like nutrient starvations, heavy metal content and high light intensities can enhance astaxathin synthesis. But, high salt concentrations can affect the cell and promote mortality at higher rate (Markou and Nerantzis, 2013). Phycobilins are coloured different proteins in microalgae, which absorbs solar radiation in the region of the visible spectrum in which chlorophyll have low absorption (Kannaujiya et al., 2018). Unlike other secondary metabolites, these protein-binded pigments show decrease in cellular levels in response to stress conditions like nutrient starvations and heavy metal exposure. But, increase in salt concentration to certain extent can elevate their production rates (Markou and Nerantzis, 2013). Thus, it is important to optimise the culture conditions to make it suitable for enhanced production of the target metabolite along with higher growth rate of the selected organism.

### Mycosporine-like Amino Acids

MAAs are water soluble photoprotective compounds with absorption maxima ranging between 309 and 360 nm (Hartmann et al., 2015). The *de novo* synthesis of MAAs can be done by microalgae, macroalgae, cyanobacteria and fungi. MAAs are recognised as the strongest UV-A absorbing molecules and in addition to it, these do not generate any Reactive Oxygen Species. Mycosporine-2- Glycine (M2G) is recently discovered MAA and is sole MAA present in *Aphanothece halophytica* (Tarasuntisuk et al., 2018). MAAs are primarily isolated and purified from fungi and the comparative higher expressions of these compounds in microalgae were identified later. They are extremely stable and have a chromophore ring with amino alcohol and glycine at the C1 and C2 positions, respectively. The shikimate and pentose phosphate pathways are the two metabolic pathways involved in MAA synthesis, with the former being the most common. The amount of MAA expressed inside the cells is associated with the amount of light and UV radiations (Raj et al., 2021). They prevent thymine dimer formation and protect the cells from DNA damage. Due to their potent photoprotectant properties, MAAs are generally referred as microbial sunscreens and MAA like shinorine are widely used in industrial level sunscreen productions (Nowruzi et al., 2020). Although, MAA are known for their photoprotective properties, only few of them are commercially produced. It is well known that UV stress can enhance MAA production, but optimization of the wavelength and exposure time is necessary in large-scale productions for best product recovery. Abiotic stress related responses and its associated genes responsible for enhanced MAA production are yet to be studied.

### Alkaloids

Alkaloids are nitrogen-containing compounds produced by cyanobacteria, microalgae, plate fungus, and other organisms. These compounds are difficult to differentiate from other naturally occurring nitrogen compounds. The majority of the metabolites are commercially valuable, but some of them may have poisonous properties, such as anatoxin, a neurotoxin that can cause paralysis and death in some fish and animals. Similarly, Spirolides are macrocyclic imines which constitute spiroacetal groups in their chemical structure. These compounds exhibit toxic properties and target acetyl-choline receptors and calcium channels (Sasso et al., 2011). Chemical derivative of alkaloid with indole ring in its structure is classified as indole alkaloids. These compounds often exhibit antimicrobial and anticancer properties (Jeong et al., 2020). In addition, drugs like morphine, quinine, papaverine and several other pharmaceutically important compounds have an alkaloid structure. Microalgae like Scenedesmus sps, Arthrospira platensis and Isochrysis galbana are some of the species which have potential in Alkaloid production and accumulation (Hadizadeh et al., 2019; Kaliwal, 2019; Fabiola et al., 2020). There are different subclasses of alkaloids and the biosynthesis pathways of molecules in each class may vary. Zooxanthellamine, an alkaloid present in dinoflagellates have structural similarity with the nitrogen compounds present in the soft corals which indicate symbiotic relationship between these organisms, where the soft corals might have acquired these compounds through food chain (Sasso et al., 2011).

### Terpenoids

Terpenoids are other diverse class of secondary metabolites, which are expressed in almost all domains of life. But, the diversity is high in plants with complex metabolic pathways and interaction with the surrounding environment. It has wide range of applications at industrial level such as in pharmaceutical, medical and food industries. For example, Betulin is a triterpenoid known for its anticancer and antiviral porperies. The biosynthesis of terpenoids involve three major steps *viz.,* synthesis of C_5_ Precursors, Polyprenyl pyrophosphate synthesis and their processing to form isoprenois end products. The well-known compounds like sequiterpene, diterpenes and monoterpenes belong to this class of metabolites (Sasso et al., 2011). Metabolic routes of terpenoid synthesis involve mevalonate (MVA) and the methyl-D-erythritol (MEP) pathways, which produce the precursors of terpenoid synthesis, 5-carbon prenyl phosphate. Metabolites like terpenoids and several others are expressed in plants with complex metabolic pathways. However, the expressions in such natural hosts will be in very low amounts and can be incorporated into photosynthetic microalgae for higher production rates. *Chlamydomons reinhardtii* and *Phaeodactylum tricornutum* are the common model organsisms used in the genetic engineering studies including the foreign gene insertions. (Vavitsas et al., 2018; D’Adamo et al., 2019). As the biosynthesis pathway, enzymes involved and precursors are known, each of them can be a targeted using genetic engineering to enhance the terpenoid production in microalgae.

Lipids and nucleosides are often thought of as primary metabolites, yet due to certain features of these compounds, they are sometimes classified as secondary metabolites. For example, *Tolypothrix tenius* produces nucleosides such as toyocamycin and tubercidin, which have antifungal properties (Jeong et al., 2020). PUFAs (polyunsaturated fatty acids) are fatty acids which can be consumed as nutritional supplements and can help to prevent certain diseases. PUFAs like eicosapentaenoic acid (EPA), docosahexaenoic acid (DHA), linolenic acid and arachidonic acid are some of the widely studied compounds in combination with industrial productions. PUFAs also serve as substrates for enzymatic oxidations and result in compounds like oxylipins (Sasso et al., 2011). Fish oil is the widely used source of PUFAs but, the primary producers are microalgae. The primary PUFAs can be changed biochemically by undergoing conversions when they pass through the food chain and this process is called as trophic upgrading (Bule et al., 2018). Similarly, sterols are lipid containing molecules and are characteristic feature of eukaryotic cells. They play a role in maintaining the physiochemical properties of the cell and constitutes about 20–30% of the membranes (Vuppaladadiyam et al., 2018). To enhance the production of these secondary metabolites for commercial applications, apart from standardizing the culture conditions, genetic engineering can play an important role. However, there are several limitations with respect to availability of genomic sequencing data for several non-model algal species as well as the standard transformation methods.

## Algal Transformation Methods

**FIGURE 1:**
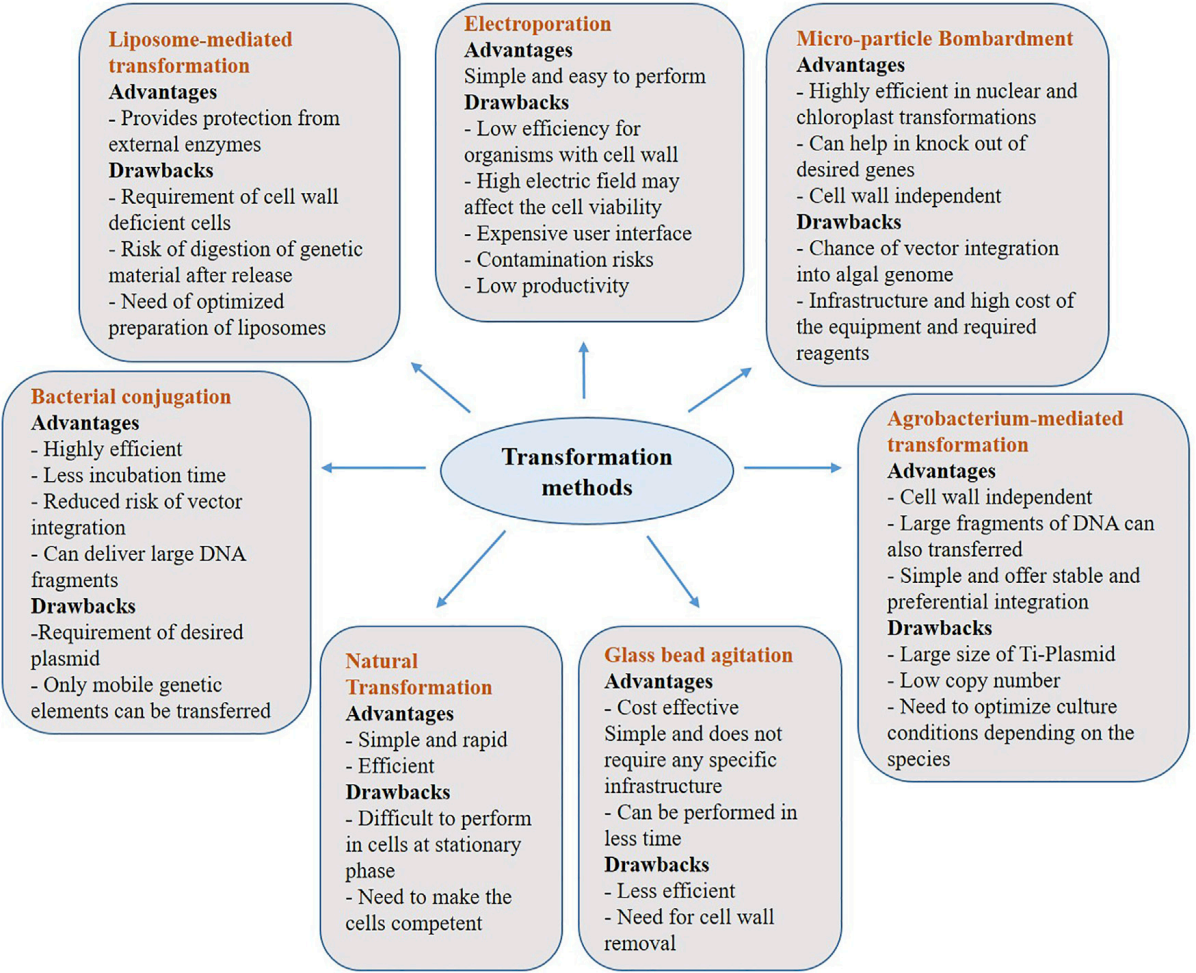
Advantages and Disadvantages of transformation methods used in algal genetic engineering. Transformation of a cell refers to integration of a desired gene at the target site. Although, genetic engineering is a highly efficient and sophisticated technique, there are certain disadvantages along with its advantages.

Microalgae can be transformed using various recombinant DNA techniques to ensure higher yields of the targeted metabolite. To alter the genetic makeup of microalgae by inserting foreign genes, different transformation techniques have been employed. Below we have described some of the key transformation methods, which proven to useful in transforming various algal strains and Figure 1 depicts advantages and disadvantages of each of the below discussed methods.

### Electroporation

One of the efficient transformation methods, where algal cells are made competent by applying electric shock for a short period of time using electrodes. This generates a voltage difference across the algal membrane that creates a temporary disturbance in the phospholipid bilayer, creating pores, which in turn allows our desired molecules (foreign DNA) to pass across the membrane (Boni et al., 2015). *Synechococcus* sp. was the first cyanobacterium transformed by electroporation, followed by the cell wall less mutants of *Chlamydomonas reinhardtii*, in which the transformation efficiency was several folds higher than glass-bead transformation (Banerjee et al., 2016). The voltage applied may affect the cell viability and to mitigate such limitations, microfluidic electroporation systems can be used to control the voltage applied. Stable gene transfers in microalgal species like *Scenedesmus obliquus*, *Nannochloropsis* sp. and *Chlorella vulgaris* have been achieved using electroporation. (Im et al., 2015).

### Microparticle Bombardment (Biolistics)

This method works on accelerated or high-speed gold/tungsten microparticles, which are coated with DNA and shot using high pressure to transform the target cells (Gutiérrez and Lauersen, 2021). This method can be easily used for the Cas9-gRNA ribonucleoprotein delivery into cells, which helps in the knockout of genes. However, the biggest concern is transgenic integration of vector DNA into the algal genome because, removing this integrated vector is quite challenging (Sharma et al., 2018). Transformation efficiency depends on cell density, DNA concentration, kinetic energy of the particles, temperature and regeneration efficiency of the cell. (Vazquez-Villegas et al., 2018).

### Agrobacterium-Mediated Transformation

Bacterium, *Agrobacterium tumefaciens* is used in this method to transfer DNA segment into the target cells by natural infection process. The T-DNA sequence (part of Ti plasmid) of the bacterium, which allows insertion of the desired DNA construct is utilized for the transformation (Pazhukunnel et al., 2015; Banerjee et al., 2016) This method has been utilized in transformation of microalgal species like *Chlamydomonas reinhardtii*, *Haematococcus lacustris*, *Chlorella* sp, *Dunaliella bardawil*, *Dunaliella salina*, *Symbiodinium* sp, *Nannochloropsis* sp. and *Parachlorella kessleri*. (Vazquez-Villegas et al., 2018). The efficiency depends on pH, temperature, culturing time, bacterial density and their virulence gene induction pattern (San et al., 2011; Gutiérrez and Lauersen, 2021). Pratheesh et al., in 2014, reported an efficient agrobacterium mediated transformation in *C. reinhardtii*, in which glycine bethaine and pH stress was utilized to induce their virulence gene (*vir* gene), which in turn increased their transformation frequency (Pratheesh et al., 2014).

### Glass Bead Agitation

In this method, microalgal cells are mixed with foreign DNA, polyethylene glycol (PEG) and glass beads of about 0.5 mm in size, followed by agitating the mixture. PEG acts as a membrane fusion agent (Fajardo et al., 2014; Vazquez-Villegas et al., 2018). Microalgae with less rigid cell wall or thin cell walls, like *Dunaliella salina* and cell wall deficient strains of *C. reinhardtii* can be efficiently transformed. Presence of cell wall, velocity and duration of the vortexing, cell size, concentration of the non-ionic surfactant used (PEG) are some of the main factors, which can affect the transformation efficiency. Agitation of the cells using silicon-carbide whiskers is also a popular method used in which the cells are vortexed using ceramic microparticles composed of silicon and carbon. Cell walled, *Amphidinium* sp, *Symbiodinium microadriaticum* and *C. reinhardtii* are also transformed using this technique. Although the transformation efficiency is much better but the health hazard caused by inhaling silicon while handling is one of the major drawback (Rathod et al., 2017).

### Natural Transformation

Microalgae and cyanobacteria are capable of DNA uptake from its surrounding environment naturally without using any surfactants (Stucken et al., 2013). Double crossover homologous recombination is the process in which the gene replacement takes place in cyanobacteria and microalgae, after natural transformation. The foreign DNA used for this procedure can be either linear or can be carried on replicative or integrative plasmids. Pili type IV and secretion system type II are two main components of these organisms, which play an important role in natural transformation. *Synechocystis* PCC 7942, *Synechococcus* PCC 7002, *Synechocystis* sp. PCC 6803 and *Thermosynechococcus elongatus* BP-1 are naturally transformable cyanobacterial species (Gutiérrez and Lauersen, 2021).

### Bacterial Conjugation

This method of transformation works on the basis of bacteria’s ability to exchange plasmids through their pili via direct contact between the donor and the recipient. This is a natural type of transformation in which combinations of shuttle, helper and conjugative plasmids are used. The main role of helper plasmid is to prevent the degradation of vector by the restriction enzymes. Helper plasmid along with shuttle plasmid mediate the conjugative plasmid transfer from the bacterium to the target cell. The main factors, which affect the transformation efficiency are the ability of the strain to maintain and integrate the vector after transformation and the growth stage of both the recipient and donor organisms, etc. (Karas et al., 2015). *Synechococcus*, *Prochlorococcus*, *N. punctiforme*, *Anabaena variabilis*, *Nostoc* PCC 7120, *S. elongatus* and *Synechocystis* sp. are some of the cyanobacterial species in which this method has been used. *Phaeodactylum tricornutum*, *Thalassiosira pseudonana*, *Acutodesmus obliquus*, and *Neochloris oleoabundans* are some of the microalgal strains transformed using this method. It was found that bacterial conjugation based CRISPR-Cas9 editing has higher efficiency than biolistic-based CRISPR-Cas9 gene editing in microalgae (Banerjee et al., 2016) An efficient and stable transformation of foreign DNA for the first time was performed in oleaginous green microalgae - *Acutodesmus obliquus* and *Neochloris oleoabundans*, via bacterial conjugation using *Escherichia coli* as the donor. In this study, the constructed conjugative plasmids for transformation consisted an origin of transfer (*oriT*), *mob* genes, which are required for the DNA mobilization from the bacterial donor to the microalgal cells, an antibiotic resistance gene, which acts as a selectable marker and the Clover gene as a green fluorescent reporter (Muñoz et al., 2019).

### Liposome-Mediated Transformation

Liposomes can be defined as neutrally charged or cationic microscopic, phospholipid vehicles, which are made of single lipid bilayers, in which the foreign DNA to be transformed is loaded in their aqueous compartment. The cationic structure of the liposomes enables encapsulation of the foreign genetic material, which helps in transformation. This helps in producing a stable complex that prevents the degradation of the genetic material (Rabinovich and Peng, 1997). Liposome mediated transformation works in such a way in which the cationic liposome complex fuses with the negatively charged membrane to deliver the foreign genetic material into them (Muñoz et al., 2019; Ohta and Ichihashi, 2019). Certain cell wall less mutants of *Chlamydomonas reinhardtii* and *Dunaliella salina* can be studied using this method.

The majority of the transformation strategies discussed above are commonly used in algal genetic engineering. However, each method has its own set of advantages and disadvantages. As a result, selecting a transformation method is an important factor in ensuring a successful transformation. Condition optimization, cell wall rigidity, and the type of genetic material to be transferred are a few of the critical aspects to considered before deciding on a method to obtain higher transformation efficiency.

### Current Approaches in Algal Genetic Engineering

**FIGURE 2:**
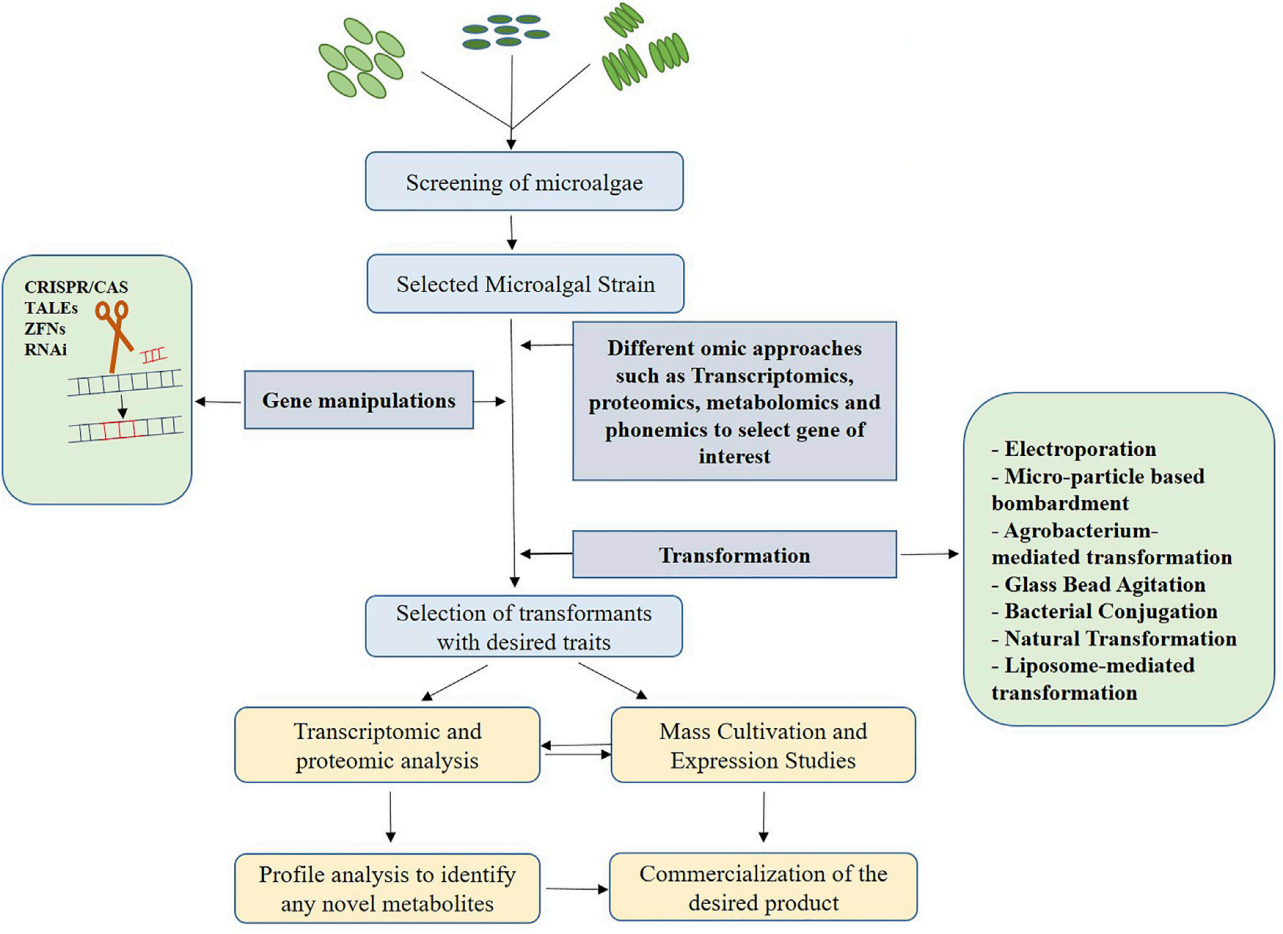
Steps involved in secondary metabolite production using genetic engineering. Primarily, the cells are screened on the basis of growth rate and higher productivity. Further, transcriptome analysis is performed to select the target, followed by designing editing tools and transformation of the gene of interest into the target site. The transformants with the desired traits selected can either be used to perform omic analysis and profile analysis to identify any novel metabolites or directly can be used for mass cultivation and expression studies. If any novel metabolites identified, the product is commercialized.

Despite the fact that microalgae are recognized for synthesizing metabolites and value-added products, the majority of them are produced in relatively small quantities. However, in order to commercialize any of the products for industrial use, the production rate must be high to meet economic demands. Random mutagenesis using a variety of physical and chemical mutagens is usually followed by screening techniques in the commercial production process (Fu et al., 2019). But, this is however unpredictable and time-consuming. The ideal strategy involves genetic and metabolic engineering involving omic technologies to identify target sequences for the study and to manipulate the respective gene sequences to yield larger quantities of the desired product. Transcriptome analysis can be used to compare sequences in order to find transcription factors and its expression levels (Bajhaiya et al., 2017), while proteomics can be used to examine post-translational responses using advanced techniques like GC-MS(Arias et al., 2020; Jeong et al., 2020) Liquid chromatography Mass Spectroscopy (LC-MS) can be employed in metabolic profiling to accurately isolate and distinguish the secondary metabolites MAAs (Hartmann et al., 2015). Figure 2 represents various steps involved in the commercial production process using genetic engineering. Up-regulation and down-regulation of transcriptional and translational genes, as well as knock-out and knock-in of the desired genes, are all part of this process. In the case of secondary metabolites, the target genes will be involved in metabolic pathway regulation, either to produce an enzyme or a product of the pathway (Saini et al., 2020).

Several industrially important secondary metabolites are naturally present in some of the plants. However, the cultivation and purification of these products from natural hosts is not cost-effective. Furthermore, they are produced in extremely small quantities and are found in complicated combinations with related molecules, making isolation difficult. As a result, research is being done to introduce product-specific genes into suitable hosts such as microalgae due to their photosynthetic properties and most importantly they do not pose any threat to environment (D’Adamo et al., 2019). Lauersen et al. (2018) expressed the plant diterpene synthases (diTPSs) into algal chloroplast and the results show over production of both the enzyme diTPSs and the diterpenoid products like casbene and taxadiene (Lauersen et al., 2018). Extra chromosomal (Episome-based transformation) genetic engineering studies for metabolic pathway engineering by Fabris et al. (2020) on *Phaeodactylum tricornutum* for expression of plant monoterpenoid Geraniol was successful in demonstration and the profiling analysis shows the maximum Geraniol titre as 0.309 mg/L (Fabris et al., 2020).

Several studies have also been performed to identify the target gene to be modified to over-produce the desired metabolite. Kato et al. (2021) performed mutational breeding and resulted in a lipid and carotenoid rich mutant, KOR1. Profile analysis of cell components and metabolome analysis were performed to study the underlying mechanism for lipid and carotenoid accumulation. Insertion/Deletion mutations in *ISA*1 gene encoding a Starch debranching enzyme was observed and the disruption of this enzyme enhanced carbohydrate degradation and repartitioning the carbon to lipid and carotenoid productions. These results suggest the debranching enzyme as a target for metabolic engineering studies (Kato et al., 2021). Similar studies can be performed targeting other key enzymes and regulators to obtain higher rates of secondary metabolite accumulations. Phytoene synthase (PSY) is a rate-limiting enzyme in carotenoid biosynthesis pathway. There are several studies supporting this, such as PSY overexpression showed increased carotenoid production and suppression resulted in reduced production. To study the effect of growth stage on carotenoid synthesis, Kadono et al. (2015) fused the *psy* gene with fluorescent protein to observe the changes in the carotenoid expression levels in *Phaeodactylum tricornutum* at different growth stages. The obtained results of the transformants were compared with the wild type and found that there was 1.45-fold increase in carotenoid content in the transformants. Further it was also concluded that the PSY mRNA levels at the log phase contributes to the increased carotenoid production in the stationary phase (Kadono et al., 2015). Such new findings will be useful large-scale cultivations, where the organism can be harvested at the right phase with maximum yield of the desired metabolite.

Multiple genes can also be transformed into a single organism. A study on single, double and triple transformation in *Phaeodactylum tricornutum* using one, two and three carotenoid biosynthesis genes respectively shows enhanced carotenoid production. The genes used in the experiment were Violaxanthin de-epoxidase (Vde), Vde-related (Vdr) and Zeaxanthin epoxidase 3 (Zep3). Studies were performed to evaluate the change in the pigment content and the results suggest that the triple transformants showed 4-fold increase in fucoxanthin content (Manfellotto et al., 2020). In the same way, multiple genes involved in a metabolic pathway can be transformed and altered to alter the biochemical pathways in order to obtain higher production rates.

Secondary metabolite accumulations in response to induced-stress conditions are quite common and well-known. But, it is equally important to understand the underlying mechanisms which causes the change in metabolite levels inside the cells. A recent multi omics study on *Chromochloris zofngiensis* by (Mao et al., 2020a) was performed to study the effect and the underlying mechanisms of TAG and astaxanthin accumulations under salt stress. This study has optimized the salt concentrations and also identified the key regulators which cause the metabolic shifts (Mao et al., 2020b). This study was also helpful in finding that *Chromochloris zofngiensis* can tolerate moderate salt stress and thus sea water can be employed in large-scale productions instead of freshwater. Another study employed time-resolved transcriptional analysis on *Chromochloris zofngiensis* to study the transitions depending on the nutritional status. The transitions were observed between sulphur starved and sulphur-replenished conditions (Mao et al., 2020a). This study was helpful in finding the role of key regulators and its effect on redirection of biochemical pathways leading to TAG and astaxathin accumulations. These regulators can be future targets for genetic engineering to produce potential mutants with higher yields. Each of the study discussed above employs specific transformation method suitable for the organism under study and further coupled with omic approaches to find the desired genes to be targeted. This is crucial as most of the metabolites are industrially important and to meet the existing demands higher production rates are required for commercialization processes.

## Industrial Applications of Algal Secondary Metabolites

Microalgae and its metabolites can be used to produce a variety of industrially important products. These metabolites can be bio-pigments, MAAs, Isoprenoids, Alkaloids etc. In comparison to synthetically available pigments, microalgal bio-pigments are renewable, safe, and effective. Humans are not capable of denovo synthesis of pigments such as carotenoids. As a result, dietary consumption of such pigments is essential to meet the requirements (Fayyaz et al., 2020). Carotenoids are pigments that have a variety of medical properties and are found in many foods, cosmetics, and pharmaceuticals. Carotenoids are powerful antioxidants with anticancer properties. Carotenoids have commercial importance due to several properties like antioxidant property, brilliant colors and health promoting factors (Chakdar and Pabbi, 2017). Major carotenoids of microalgae are astaxanthin, lutein and β-carotene. Other molecules like polyketides, phenolic compounds, polyhydroxyalkanoates, proteins and peptides also exhibit strong antioxidant characteristics. These are produced in high amounts to protect the cells from hydrogen peroxide and oxygen radicals produced due to oxidative stress (Vuppaladadiyam et al., 2018). The carotenoids leutin and zeaxanthin are found in the eye as protecting macular pigments. Studies have shown that, consuming more carotenoids through diet lowers the risk of age-related macular degeneration (Wu et al., 2015; Rosales-Mendoza, 2016). Further the β-carotene also known as orange carotenoid can functions as a precursor for Vitamin-A, a vitamin that is involved in important physiological processes involving lung development, tissue maintenance and regeneration and can cause serious disorders such as disturbing the normal lung functioning leading to tissue dysfunction, if deficient (Timoneda et al., 2018). Phytoene and β -Cryptoxanthin are carotenoids with anti-inflammatory properties (Mason et al., 2015). Consumption of metabolites like phenolics through dietary supplements can reduce the risk of certain degenerative disorders such as Alzheimer’s and Parkinson’s diseases.(Machu et al., 2015; Deviram et al., 2020).

Species like *Chlorella zofingiensis, Chlorella vulgaris, Dunaliella salina* and *Haematococcus pluvialis* are common commercial producers of carotenoids*. Haematococcus pluvialis* is a commercial producer of astaxathin. Large-scale cultivation of the organism is usually performed employing a two-stage cultivation system, where the cells are grown at optimum conditions in the first stage and later subjected to combinations of stress conditions for enhanced pigment accumulations (Markou and Nerantzis, 2013). Phycobilins have a good economic value due to their high coloration effects. They are used as biochemical tags, food colorants and in the cosmetics industries (Kannaujiya et al., 2018).

A terpenoid called Betulin is extracted from algal species like *Phaeodactylum tricornutum* with the potential to act against HIV and certain cancers such as lung cancer, colorectal cancer and cervical cancer (Schwiebs and Radeke, 2018; D’Adamo et al., 2019; Zehra et al., 2019). Important amino acids such as MAAs are also extracted from algal species such as *Chlamydomonas hedleyi, Alexandrium* sps and *Gymnodinium catenatum* (Raj et al., 2021). MAA are photoprotective compounds with wide range of applications due to their anti-inflammatory, antioxidant, and anticancer properties. Osmoregulation and wound healing properties are also reported from MAAs (Choi et al., 2015; Gharib et al., 2020). The MAAs can act against cancer cells by blocking their proliferation or by triggering apoptosis. When it comes to wound healing, MAAs stimulate a number of factors such as Focal Adhesion Kinases (FAKs) and Extracellular signal Regulated Kinases (ERKs)and activates the signalling pathways that help in the healing process (Raj et al., 2021).

Silver Nanoparticles are commonly employed in cancer treatment to induce cytotoxicity in cancer cells, but non-cancer cells are frequently damaged as well. To lessen such negative effects of nanoparticle application, combination of metabolites from microalgal crude extract could benefit theranostics (Hussein et al., 2020) by limiting the damage to non-cancerous cells. Microalgal crude extracts can be made with a variety of solvents, including water, ethanol, diethylether, and others, to study their antibacterial and anticancer properties. Similarly, Alkaloids like sytonemin and hapalindole also have commercial importance. Sytonemin is photoprotective in nature while hapalindole has antibacterial, antituberculosis and anticancer properties (Jeong et al., 2020), which can have several applications starting from cosmetic industries to human therapeutics. Similarly the exopolysaccharides from microalgae such as *Synechocystis* and *Gloeocapsa* sp. have antipathogenic effect against food borne pathogens (Najdenski et al., 2013) and has been tested to control the growth of *Staphylococcus aureus, Escherichia coli* and *Bacillus cereus*. *Scenedesmus* species are commonly studied to explore the medicinal properties of their metabolites due to their higher growth rate and ease to harvest. Marrez et al. (2019) investigated the anticancer and antimicrobial properties of *Scenedesmus* crude extract and its fractions. The findings show that both the crude extract and its fractions have a substantial antibacterial and antifungal activity against the bacteria and fungi such as *Salmonella typhii, Pseudomonas aeruginosa, Aspergillus steynii* and *Aspergillus carbonarius*. These chemicals also showed cytotoxicity against human breast MCF7, hepatic HepG2 and colon HCT116 cancer cell lines (Marrez et al., 2019) which indicates the possible wider application in cancer treatments.

Algae derived lipid containing molecules such as PUFAs and sterols have high commercial importance due to their health benefits and potential applications in food industries. PUFAs are used in treatment for certain diseases such as atherosclerosis, Alzheimer’s, psoriasis and rheumatoid arthritis (Bule et al., 2018). Algae like *Thraustochytrium* sp. and *Parietochloris incisa* are well recognized as industrial PUFA producers, which can accumulate high amounts of lipids under stress conditions especially in nitrogen deplete medium. Microalgae have a highly diverse sterol profiles, which include phytosterols, fucosterols and stenols. Phytosterols have been reported with properties like hepatoprotective, immunomodulatory, anti-inflammatory, anticancer, antioxidative and also can regulate high cholesterol levels (Vuppaladadiyam et al., 2018). Shanab et al. (2012) studied the effects of aqueous extracts of microalgae for its antioxidant and anticancer properties. The results show an antioxidant efficiency ranging from 32 to 76% and anticancer efficiency of 89.4% when tested in human hepatocellular cancer lines. Among the cultured strains, *Nostoc* and *Oscillatoria* sps. Showed high increase in the phycobilin content followed by increased antioxidant and anticancer properties (Shanab et al., 2012), which suggest that cyanobacterial diversity can also be a potential source for bioactive compounds.

The chemical-based formulations of cosmetics have several side-effects,which may cause hypersensitivity and anaphylactic reactions. These negative effects can be tackled by using microalgal metabolites as a promising alternative source of natural cosmetics. They are bio-compatible and are of high demand in cosmetic productions. This is due to their anti-blemish, antimicrobial, anti-aging, anti-inflammatory, UV protectant and skin whitening properties. Microalgal bio-pigments can be used as natural colouring agents in products like shampoo, soap, eye-shadow, lipsticks and lotions. Bioactive compounds from *S. plantensis* have vital role in skin repair and help in preventing skin wrinkles (Gürlek and Oncel, 2020). Extracts from *Chlorella vulgaris* reported to promote tissue regeneration by stimulating collagen synthesis (Kumar et al., 2018). However the aging can be caused by many of the internal and external factors. The internal factors can be hormonal or genetic disorders and external factors affecting aging are exposure to UV and toxins or less care and moistening given to skin. These factors cause many changes to skin like formation of Reactive Oxygen Species (ROS), Advanced Glycation Endproducts (ADE) and Matrix Metallo Proteinases (MMPs) (Gürlek and Oncel, 2020). M2G, a rare MAA is recently known for its inhibitory effect on AGE formation and has potential to be used in anti-aging formulations. M2G accumulations can be enhanced by UV irradiations and salt stress. This indicates that M2G has both photoprotectant and osmoprotecant properties. It is a free radical scavenger and also can act against oxidative stress induced melanoma in humans (Tarasuntisuk et al., 2018).

Microalgae can also produce metabolites with potential antiviral properties. The demand for pharmaceutical industry is increasing as the severity of viral outbreaks increases. In particular, COVID 19 pandemic has had a significant impact, and substantial research is being conducted to combat the virus. However, the majority of investigations are conducted in common expression systems such as mammalian cells, which pose a significant risk of pathogen contamination. Algae can be used to produce and potentially deliver important biopharmaceuticals to tackle this problem (Rosales-Mendoza et al., 2020). Recent studies suggest that algae can also be employed to produce vaccine, serological kits, nasal sprays and respirators and act effectively against SARS-COV-2 (Chia et al., 2021). Human therapeutic proteins can also be expressed in algal cells with significant expression levels by recombinant technology. Furthermore, the produced proteins are bioactive and can be incorporated and expressed in the chloroplast genome for better expression (Rasala and Mayfield, 2015). The algal biomass or extracts showed immense potential to be used in biopharmaceutical and nutraceutical industries however in spite of that the utilization of algal extracts at industrial level is still limited. The production cost and the quantity of metabolites generation is a challenge, which can be counter with the help of genetic engineering. Although microalgae have a faster growth rate and higher productivity, genetic engineering technologies can aid in greater accumulations of the desired product. However, this comes with its own set of challenges and limitations.

## Challenges and Limitations in Algal Genetic Engineering

Although algal genetic engineering is known for enhanced metabolite productions, it also has certain challenges and limitations. Firstly, the challenge lies in the biochemical pathways that are not very well known in microalgae. Moreover, metabolic drifts may occur resulting in a partial conservation in the process of evolution. In addition to it, genome manipulations in microalgae are quite challenging due to their complex cellular structures and for the same reason they are underexplored compared to simple organisms like bacteria. Moreover, the genome sequences are available only for few species. One of the whole genome sequenced organism being *Chlamydomonas reinhardtii,* which is considered as a model organism for molecular studies. This model organism has been studied for essential cellular processes like photosynthesis, light perception and engineering studies for enhanced metabolite productions. But, it has not yet been used for industrial productions (Chakdar et al., 2021). Recently, there is progress in genetic engineering and transformation studies in species other than model organisms but, all the transformation methods are not well studied in microalgae. Moreover, the efficiency of the transformation depends on the selection of suitable gene transfer method. This is because the cell wall composition of algae varies from species to species and the organisms with rigid cell wall is difficult to perform gene deliveries. Different parameters can be changed to increase the permeability of the cell wall, but the product recovery and efficiency will be very less.

Transformation efficiency also depends on the organism under study. For example, *Haematococcus lacustris* is well recognised as an excellent astaxanthin producer, but the efficiency of transformation is less due to certain features like its slow growth rate, sensitivity to stress and its thick cell wall (Tran and Kaldenhoff, 2020). After transformation, the next challenge lies in the selection of transformants, which is commonly performed using selectable markers, which confers a survival advantage. Insertion of antibiotic resistance genes is a widely used method for selection of transformants. The antibiotics should be chosen considering the native habitat of the microalgae under study. Marine microalgae transformant selection has an additional challenge because high salt concentrations reduce the antibiotic activity and certain antibiotics like streptomycin and kanamycin cannot be used even in low salt concentrations (Vazquez-Villegas et al., 2018). Following transformant selection, transcriptomic and proteomic analysis to compare it with the wild type is carried out. But, there might be a difference in the comparative data between these analyses. This is because, not every gene in the mRNA is translated into protein or a single gene can code for multiple proteins.

In algal genome editing, the foreign gene insertion can be done either into the nuclear genome or into the chloroplast genome. Chloroplast expressions have shown higher and a broad range of metabolite accumulations compared to nuclear genome transformations. But, among the successful transformations, only few proteins accumulate and others do not. This may be due to the susceptibility of the recombinant proteins to proteases or the folds of mRNA which blocks the translation initiation. Other limitation is that the proteins expressed in chloroplast are not secreted and the cells must be lysed to isolate the metabolite followed by purification (Rasala and Mayfield, 2015). All these challenges can be tackled to some extent with specific strategies.

## Future Perspectives

Microalgal engineering has progressed in recent decades, with the majority of studies focusing on primary metabolites, particularly lipid metabolism. Algal secondary metabolites should be explored in every sector, with further research being performed to overcome the challenges using advanced strategies. Until now, traditional engineering, which involves random insertion into the host genome followed by screening is the widely used strategy in applied research. Target specific gene editing has become popular in recent years as a result of the development of powerful gene editing technologies. Advanced techniques can act on critical enzymes, which are involved in the rate limiting steps. Such techniques include Transcription Activator Like Receptors (TALEs), Clustered Regularly Interspaced Short Palindromic Repeats (CRISPR), Zinc Finger Nucleases (ZFNs), etc. which can alter the metabolite production rates at higher rates. RNAi and riboswitch engineering are also efficient in microalgal research.

Along with the advancements in gene editing techniques, sequencing technologies should also improve to make sequences more accessible and to study non-model organisms that are industrially important. Further, advanced bioinformatics approach in combination with genomic and metabolomic data analysis should progress to study the metabolic drifts. Such drifts may cause due to the non-orthologous gene displacement or a change in the main enzyme resulting in the same phenotype (Belcour et al., 2020). These drifts are naturally occurring, but can be a strategy in future to redesign a metabolic pathway to produce multiple enzymes involved in the targeted pathway. Artificial Intelligence (AI), which has recently acquired prominence in the field of microalgal research, can be used to make molecular predictions with respect to gene sequencing and editing. AI and microalgal informatics have the potential to improve current approaches to study genetic information while can also bridge the gap in microalgal engineering (Yong et al., 2020).

The gene delivery systems are critical for successful transformation of microalgal cells to insert foreign genes. The chosen system must be appropriate for the organism being studied, which is selected depending on the cell wall composition. Recent research has found the ideal approaches for algal cells with hard cell walls, such as the zinc oxide nanowire array microdevice system, are much more efficient than conventional transformation methods (Guihéneuf et al., 2016). Algal nuclear and chloroplast engineering in combination with synthetic biology can help in commercialising algal secondary metabolites with therapeutic importance. As microalgae are free of infectious agents, most of them remain biocompatible and have several advantages over common expression systems. These features of algae can provide a scope in heterologous production of algae-based vaccines (Kumar et al., 2020).

Plants are more complicated than algae, with advanced metabolic pathways capable of producing a variety of biotechnologically relevant metabolites. However, gene editing studies are difficult to conduct due to their complexity. To obtain higher yields of the plant metabolites, respective genes can be transformed into organisms with higher growth rate like microalgae. The genes can be inserted into either the nuclear or chloroplast genomes. Chloroplast transformation results in a larger accumulation of recombinant proteins in algae and plants than nuclear transformation (Rasala and Mayfield, 2015). Optimised gene design combined with codon optimization and synthetic intron spreading can be employed to combat the low levels of expression in the nuclear transformation (Lauersen et al., 2018).

All the above mentioned strategies are mostly performed on only a few organisms for commercial product productions due to the ease of cultivation and harvesting of some common species. There is a need for more research to be performed to explore the potential of other species as well.

## Conclusion

Microalgae have the ability to produce a variety of secondary metabolites of commercial importance. Using genetic engineering, large-scale microalgae production can be made feasible. There are several studies performed on algal gene manipulations which resulted in higher yields of the target metabolites. The new technologies, when coupled with metabolic, genetic engineering methodologies and optimised culture conditions will help to improve metabolite production efficiency. In addition to it, accurate metabolic profiling also should progress to explore several secondary metabolites of industrial importance, whichin remain undiscovered till today. The proper handling and disposal of residual biomass can make industrial production of genetically altered microalgae eco-friendly.
